# Synaptonemal complex protects double-Holliday junctions during meiosis

**DOI:** 10.1101/2024.09.14.613089

**Published:** 2024-09-15

**Authors:** Shangming Tang, Jennifer Koo, Mohammad Pourhosseinzadeh, Emerald Nguyen, Natalie Liu, Christopher Ma, Hanyu Lu, Monica Lee, Neil Hunter

**Affiliations:** 1 Howard Hughes Medical Institute, University of California Davis, Davis, CA, USA.; 2 Department of Microbiology & Molecular Genetics, University of California Davis, Davis, CA, USA.; 3 Department of Biochemistry & Molecular Genetics, University of Virginia, Charlottesville, VA, USA.; 4 Department of Molecular & Cellular Biology, University of California, Davis, Davis, CA, USA.

## Abstract

Chromosomal linkages formed through crossover recombination are essential for accurate segregation of homologous chromosomes during meiosis^1^. DNA events of recombination are spatially and functionally linked to structural components of meiotic chromosomes^2^. Imperatively, biased resolution of double-Holliday junction (dHJ) intermediates into crossovers^3,4^ occurs within the synaptonemal complex (SC), the meiosis-specific structure that mediates homolog synapsis during the pachytene stage^5,6^. However, the SC’s role in crossing over remains unclear. Here we show that SC promotes crossover-specific resolution by protecting dHJs from unscheduled and aberrant resolution. When key SC components are conditionally inactivated during pachytene, dHJs are resolved into noncrossover products by Sgs1-Top3-Rmi1 (STR), the yeast ortholog of the human BLM complex^7^. Cohesin, the core component of SC lateral elements, plays a primary role in chromatin organization and is required to maintain both SCs and crossover recombination complexes (CRCs) during pachytene. SC central region component Zip1 is required to maintain CRCs even when dHJs are stabilized by inactivating STR. Reciprocally, SC stability requires continuous presence of CRCs, an unanticipated interdependence with important implications for SC dynamics. In conclusion, through hierarchical and interdependent functions of its key components, the SC enables crossover-specific dHJ resolution and thereby ensures the linkage and segregation of homologous chromosomes.

During meiotic prophase I, cohesin complexes connects sister-chromatids and mediates their organization into linear arrays of chromatin loops tethered to a common axis^2,5,8^. These cohesion-based axes define interfaces for the pairing and synapsis of homologous chromosomes that culminates in the formation of synaptonemal complexes (SCs), tripartite structures comprising the two juxtaposed homolog axes, now called lateral elements, connected by a central region of densely packed transverse filaments^5,6^ (see Extended Dats Fig. 8a). Meiotic recombination facilitates pairing and SC formation between homologous chromosomes, and then connects them via crossing over, which is necessary for accurate segregation during the first meiotic division^1^. To this end, the DNA events of recombination are physically and functionally linked to underlying chromosome structures^2^. The protein complexes that catalyze DNA double-strand breaks (DSBs) and subsequent strand exchange are tethered to homolog axes. Ensuing joint-molecule intermediates and their associated recombination complexes interact with the SC central region. A subset of recombination events is assigned a crossover fate with a tightly regulated distribution, ensuring that each chromosome pair receives at least one^2^. At designated sites, nascent joint molecules mature into double Holliday junctions (dHJs) and undergo biased resolution specifically into crossovers^3,4^, all within the context of the SC central region and associated crossover recombination complexes (CRCs). The role of the SC in crossing over remains unclear, particularly whether it plays a role after dHJs have formed to facilitate crossover-specific resolution.

## Cohesin is required for crossover-biased dHJ resolution

**Extended Data Fig. 1.**
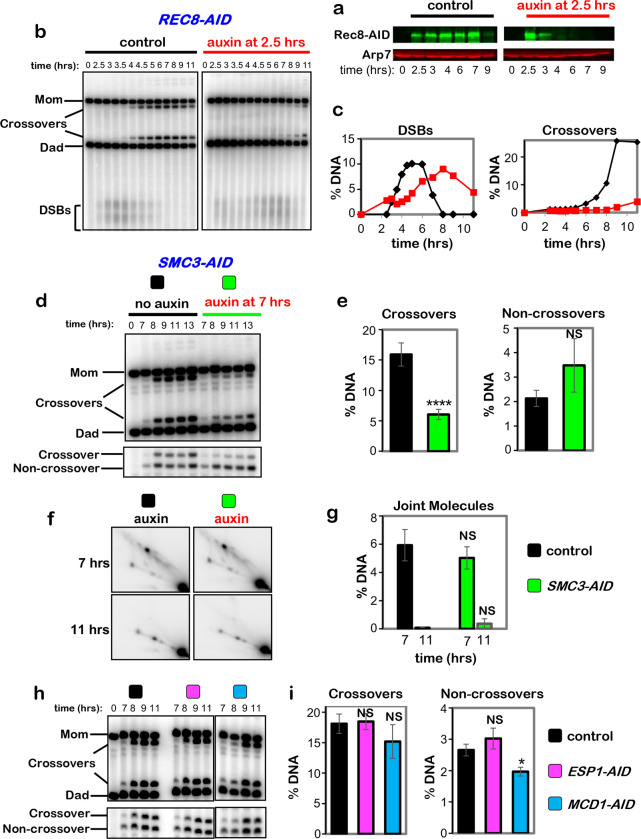
Early functions of Rec8 and roles of Smc3, Kleisin Mdc1^Rad21,^ and Separase Esp1 in crossover-specific dHJ resolution. **a,** Western analysis of early Rec8-AID degradation following addition of auxin at 2.5 hrs. **b,** 1D-gel Southern analysis of DSBs and crossovers following early degradation of Rec8-AID. **c,** Quantification of the DSB and crossover level from the Southern blot shown in C. **d,** Representative 1D-gel Southern analysis of crossover (upper panel) and non-crossover (lower panel) formation from *SMC3-AID* subcultures with or without auxin. **e,** Quantification of final levels of crossovers and non-crossovers at 13 hrs from *SMC3-AID* subcultures with or without auxin (mean ± SD, 4 independent experiments, ****P<0.0001, NS, not significant *P*=0.0561, two-tailed unpaired *t*-test). **f,** Representative 2D gel Southern analysis of joint molecules from *SMC3-AID* subcultures with or without auxin, before and after release from arrest at 7 and 11 hrs, respectively. **g,** Quantification of total joint molecule levels from experiments represented in panel **e** (mean ± SD, 3 independent experiments, NS, not significant, *P*=0.3167 for levels at 7hrs; NS, not significant, *P* >0.9999 for levels at 11hrs; two-tailed unpaired *t*-test). **h,** Representative 1D-gel Southern analysis of crossover (upper panel) and non-crossover (lower panel) formation in control, *ESP1-AID* (with auxin) or *MCD1-AID* (with auxin) strains. **i,** Final levels of crossovers and non-crossovers at 11 hrs from the indicated strains (mean ± SD, 3 independent experiments, *P=0.0146; NS, not significant, *P*=0.9610 (crossovers, control vs. *ESP1-AID*), *P*=0.1793 (crossovers, control vs. *MCD1-AID*), *P*=0.2073 (noncrossovers, control vs. *ESP1-AID*), Dunnett’s multiple comparisons test, control).

**Fig. 1.**
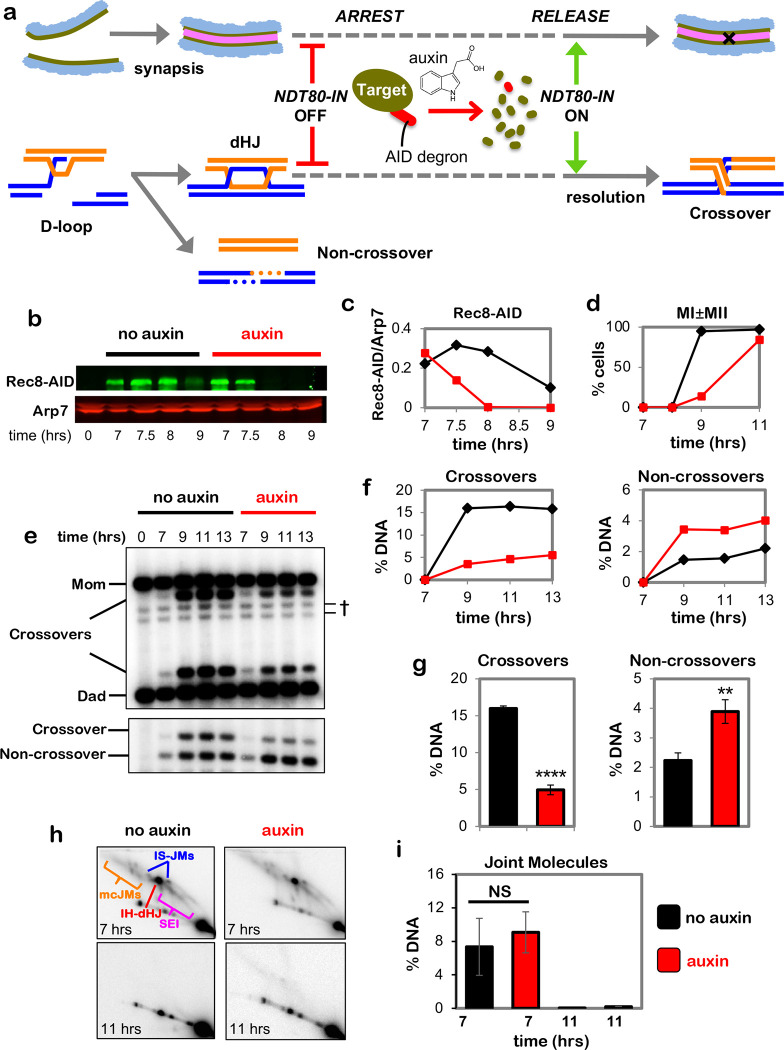
Rec8-cohesin is required for crossover-specific dHJ resolution. **a,** Experimental strategy. Top row: homolog synapsis with chromatin in blue, green homolog axes, and pink SC central region. The **X** in the final cartoon indicates a crossover formed after Ndt80 is expressed. Middle row: cell synchronization at the dHJ resolution transition using the inducible *NDT80-IN* allele; and conditional degradation of target proteins using the AID system. Bottom row: DNA events of meiotic recombination. Only the two chromosomes engaged in recombination are shown. **b,** Western analysis of Rec8-AID from subcultures with or without the addition of auxin at 7 hours. Arp7 is used as a loading control. **c,** Quantification of Rec8-AID level from the experiment shown in panel **b**. **d,** Quantification of nuclear divisions (MI±MII, cells with two and four nuclei) from *REC8-AID* subcultures with or without auxin. **e,** Representative 1D-gel Southern analysis of crossover (upper panel) and non-crossover (lower panel) formation at the *HIS4::LEU2* recombination hotspot from *REC8-AID* subcultures with or without auxin. ^†^ cross-hybridizing background bands. **f,** Quantification of crossovers and non-crossovers levels from the experiments shown in panel **e**. **g,** Quantification of final levels of crossovers and non-crossovers at 11 hrs from *REC8-AID* subcultures with or without auxin (mean ± SD, 3 independent experiments, ****P<0.0001, **P=0.0038. two-tailed unpaired *t*-test). **h,** Representative 2D gel Southern analysis of joint molecules from *REC8-AID* subcultures with or without auxin, before and after release from arrest at 7 and 11 hrs, respectively. The upper left panel highlights joint-molecule species: SEI, single end invasion; IH-dHJ, inter-homolog double Holliday junction; IS-JMs, inter-sister joint molecules; mc-JMs, 3- and 4-chromatid joint molecules. **i,** Quantification of total joint molecule levels from experiments represented in panel **h** (mean ± SD, *n=*3 independent experiments, NS, not significant, *P*=0.4561, two-tailed unpaired *t*-test).

To circumvent early meiotic defects of cohesin mutants in the formation and processing of DSBs (Extended Data Fig. 1a–d)^9,10^, the auxin-inducible degron (AID) system^11^ was used to conditionally inactivate Rec8 (the meiosis-specific Kleisin subunit of cohesin) precisely at the time of dHJ resolution (Fig. 1a). Cell cultures were synchronized at the resolution transition using an estradiol-inducible allele of *NDT80* (*NDT80-IN*) that reversibly arrests cells at the pachytene stage, in which chromosomes are fully synapsed and dHJs are poised for resolution ^12^. Auxin and estradiol were added simultaneously to degrade Rec8-AID while releasing cells from pachytene arrest and triggering dHJ resolution (Fig. 1b–d). Without auxin, Rec8-AID levels remained high until cells had completed meiotic divisions (MI±MII); but with auxin, Rec8-AID was completely degraded within 60 mins and meiotic divisions were delayed by ~2 hrs (Fig. 1c and 1d).

Crossing over at the *HIS4::LEU2* DSB hotspot^13^ was reduced by 70% following Rec8-AID degradation, accompanied by a 43% increase in noncrossover products (Fig. 1e–g). This complementary change in crossover and noncrossover levels, and the comparable kinetics of product formation with and without auxin (Fig. 1e and 1f), suggest that dHJs are efficiently resolved when Rec8-AID is degraded but their crossover fate is lost. This inference was confirmed by 2D-gel electrophoresis and Southern blotting (Fig. 1h and 1i). Thus, Rec8-based cohesin is required after dHJ formation to facilitate crossover-specific resolution. Degradation of core cohesin subunit, Smc3, confirmed that the cohesin complex, not just Rec8, is required (Extended Data Fig. 1e–h). However, the mitotic Kleisin, Mcd1^RAD21^, is not required for crossover-specific dHJ resolution indicating that this function is specific to Rec8-cohesin (Extended Data Fig. 1i,j). We also showed that separase, Esp1, does not influence crossover-specific resolution (Extended Data Fig. 1i,j), consistent with Yoon et al. ^14^ who showed that a separase-resistant allele of Rec8 does not affect crossing over.

## Cohesin and Smc5/6 function in distinct resolution pathways

**Fig. 2.**
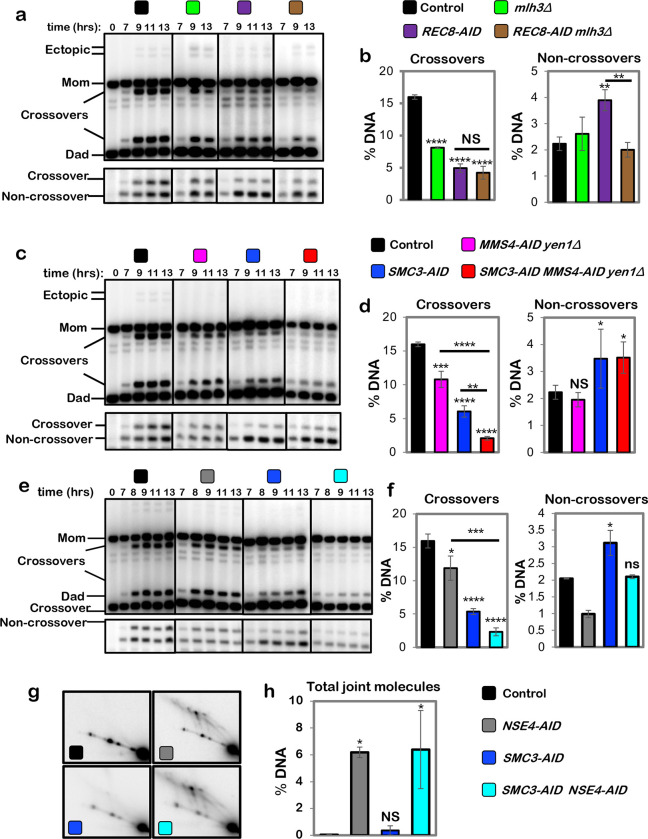
Rec8-cohesin and Smc5/6 define distinct pathways of joint-molecule resolution. **a,** Representative 1D-gel Southern analysis of crossover (upper panel) and non-crossover (lower panel) formation in control (*REC8-AID* no auxin), *mlh3*Δ, *REC8-AID* (with auxin) and *REC8-AID mlh3*Δ (with auxin) strains. **b,** Final levels of crossovers and non-crossovers at 13 hrs from the indicated strains (mean ± SD, 3 independent experiments). Statistical comparisons with control unless indicated. Dunnett’s multiple comparisons test, ****P<0.0001, NS, not significant *P*=0.5281, **P=0.0034 (*REC8-AID* vs. control), **P=0.0026 (*REC8-AID* vs. *REC8-AID mlh3*Δ ). **c,** Representative 1D-gel Southern analysis of crossover (upper panel) and non-crossover (lower panel) formation in control, *MMS4-AID yen1*Δ (with auxin), *SMC3-AID* (with auxin) and *SMC-3AID MMS4-AID yen1*Δ (with auxin) strains. **d,** Final levels of crossovers and non-crossovers at 11 hrs from the indicated strains (mean ± SD, 3 independent experiments. Statistical comparisons with control unless indicated. For crossovers, Tukey’s multiple comparisons test was performed; for non-crossovers, Dunnett’s multiple comparisons test was performed. ****P<0.0001, ***P=0.0003, **P=0.0066, *P= 0.0385 (control vs. *SMC3-AID*), *P=0.0488 (control vs. *SMC3-AID MMS4-AID yen1*Δ.), NS, not significant *P*=0.7806). **e,** Representative 1D-gel Southern analysis of crossover (upper panel) and non-crossover (lower panel) formation in control, *NSE4-AID* (with auxin), *REC8-AID* (with auxin) and *REC8-AID NSE4-AID* (with auxin) strains. **f,** Final levels of crossovers and non-crossovers at 11 hrs from the indicated strains (mean ± SD, 3 independent experiments). Statistical comparisons with control unless indicated. For crossovers, Tukey’s multiple comparisons test was performed; for non-crossovers, Dunnett’s multiple comparisons test was performed. ****P<0.0001, ***P=0.0003, *P= 0.0262 (control vs, *NSE4-AID* for crossover comparison), *P= 0.0464 (control vs. SMC3-AID for non-crossover comparison), NS, not significant, *P*=0.1148 (control vs. *NSE4-AID*), *P* >0.9999 (control vs. *SMC3-AID NSE4-AID*). **g,** Representative 2D gel Southern analysis of joint molecules from control, *NSE4-AID* (with auxin), *SMC3-AID* (with auxin) and *SMC3-AID NSE4-AID* (with auxin) strains. **h,** Quantification of total joint-molecule levels from the indicated strains. (mean ± SD, 3 independent experiments). Dunnett’s multiple comparisons test with control: *P=0.0164 (control vs. *NSE4-AID*), *P=0.0097 (control vs. *SMC3-AID NSE4-AID*, NS, not significant, *P*=0.9954).

Two classes of meiotic crossovers are distinguished by their dependencies on joint-molecule resolving enzymes: class-I crossovers depend on the crossover-specific dHJ resolvase defined by the endonuclease MutLγ (Mlh1-Mlh3)^3,4,15–17^; while minority class-II crossovers require structure-selective endonucleases, primarily Mus81-Mms4^Eme1 3,18–20^. Epistasis analysis revealed that cohesin and MutLγ act in the same resolution pathway (Fig. 2a,b); while Mus81-Mms4^Eme1^ acts in a parallel pathway (Fig. 2c,d, in these experiments Mms4-AID was degraded at the resolution transition in a *yen1*Δ background in which backup resolvase Yen1 was deleted). Mus81-Mms4^Eme1^ works in conjunction with a second SMC complex, Smc5/6 ^19–22^. Consistently, degradation of Nse4-AID (an essential subunit of Smc5/6) at the resolution transition reduced crossing over to the same extent as when Mus81-Mms4^Eme1^ was inactivated by degrading Mms4-AID (Fig. 2e–f). However, while noncrossovers were not reduced when Mus81-Mms4^Eme1^ was inactivated, Nse4-AID degradation also reduced noncrossovers 2.1-fold (Fig. 2e and 2f), indicating that Smc5/6 controls an additional, Mus81-Mms4^Eme1^-independent pathway of noncrossover formation most likely by regulating a population of the Sgs1-Top3-Rmi1 complex (discussed below).

**Extended Data Fig. 2.**
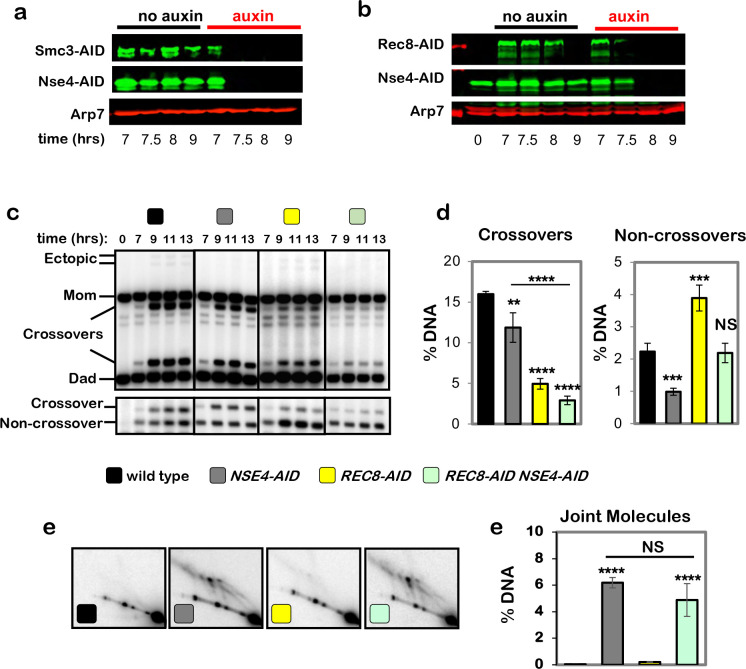
Cohesin and the Smc5/6 complex act in parallel pathways of joint-molecule resolution. **a,** Western blot images of Smc3-AID Nse4-AID co-degradation following auxin addition at 7 hours (corresponds to experiments in Fig2. e–g). Arp7 is a loading control. **b,** Western blot images of Rec8-AID Nse4-AID co-degradation following auxin addition at 7 hours. **c,** Representative 1D-gel Southern analysis of crossover (upper panel) and non-crossover (lower panel) formation in control, *NSE4-AID* (with auxin), *REC8-AID* (with auxin), and *NSE4-AID REC8-AID* (with auxin) strains. **d,** Final levels of crossovers and non-crossovers at 13 hrs from experiments represented in **b** (mean ± SD, 3 independent experiments). Statistical comparisons with control unless indicated. Dunnett’s multiple comparisons test, ****P<0.0001, **P=0.0049 (*NSE4-AID* vs. control), ***P=0.0002 (*REC8-AID* vs. control), ***P=0.0022 (*NSE4-AID* vs. control), NS, not significant *P*=0.9955). **e,** Representative Southern blot 2D gel images at 11 hours from no auxin control, *NSE4-AID* (with auxin), *REC8-AID* (with auxin) and *REC8-AID NSE4-AID* (with auxin). (e) Quantification of total joint-molecule levels from the experiments represented in **d** (mean ± SD, 3 independent experiments). NS, not significant, *P*=0.3320 Tukey’s multiple comparison test.

Confirming that Smc5/6 and cohesin act in independent pathways of resolution, simultaneous degradation of Nse4-AID and Smc3-AID resulted in an additive reduction in crossing over; and noncrossover levels that are the sum of those observed when Nse4-AID and Smc3-AID were degraded individually (Fig. 2e and 2f; co-degradation of Nse4-AID and Rec8-AID gave analogous results, Extended Data Fig. 2). Further distinguishing these two pathways, a subset of joint molecules remained unresolved when Nse4-AID was degraded alone, while resolution remained efficient when cohesin was inactivated (via degradation of either Rec8-AID or Smc3-AID; Figs. 1g,h and 2g,h and Extended Data Fig. 2); that is, Smc5/6 is essential for the resolution of a subset of joint molecules into both crossovers and noncrossovers, while Rec8-cohesin specifically promotes crossover-specific dHJ resolution, but is not required for resolution *per se* (see Extended Data Fig. 8c).

## Rec8-cohesin is required to maintain synapsis and crossover recombination complexes during pachytene

**Fig. 3.**
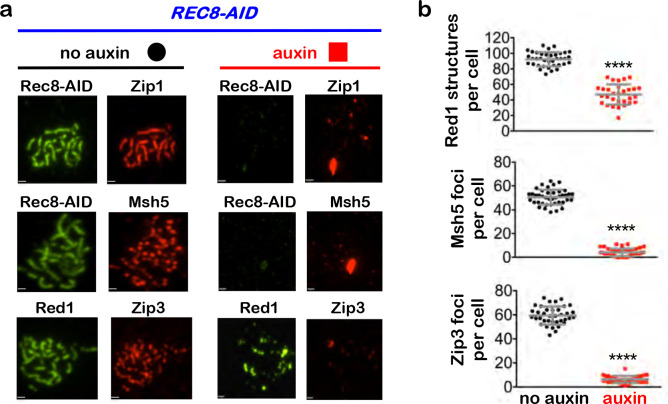
Rec8-cohesin is required to maintain synaptonemal complexes and crossover-specific recombination complexes. **a,** Representative images of surface-spread meiotic nuclei from pachytene-arrested *REC8-AID* cells with or without the addition of auxin, immunostained for the indicated markers. Scale bars = 1 μm. **b,** Quantification of Red1, Msh5 and Zip3 immunostaining structures from experiments represented in **a**. 40–60 nuclei were counted in each case. Error bars represent SD. Unpaired two-tailed *t* test. ****P<0.0001.

To begin to understand how cohesin facilitates crossover-specific dHJ resolution, Rec8-AID was degraded while maintaining pachytene arrest (no estradiol) and chromosomes were analyzed by immunostaining for markers of homolog axes (Rec8 and Red1), SC central region (Zip1), and crossover recombination complexes (CRCs; Msh5 and Zip3)^23^(Figure 3A). In no-auxin controls, linear Rec8 and discontinuous Red1 staining structures localized along synapsed homologs (indicated by lines of Zip1 staining) that were decorated with foci of Msh5 and Zip3 (Fig. 3a). One hour after auxin addition, Rec8 structures were lost and characteristic features of pachytene chromosomes disappeared: SCs disassembled and Zip1 staining was now confined to small foci and larger structures resembling polycomplexes that are diagnostic of defective synapsis; numbers of Red1 structures were reduced 2-fold, consistent with cohesin’s core function in organizing homolog axes^24^; and CRCs dissociated indicated by loss of Msh5 and Zip3 foci (Fig. 3a and 3b). Thus, cohesin is required to maintain the integrity of pachytene chromosome structures including homolog axes, the SC central region, and CRCs.

## Interdependent functions of synaptonemal complex and crossover recombination complexes are required for crossover-specific dHJ resolution

**Figure 4.**
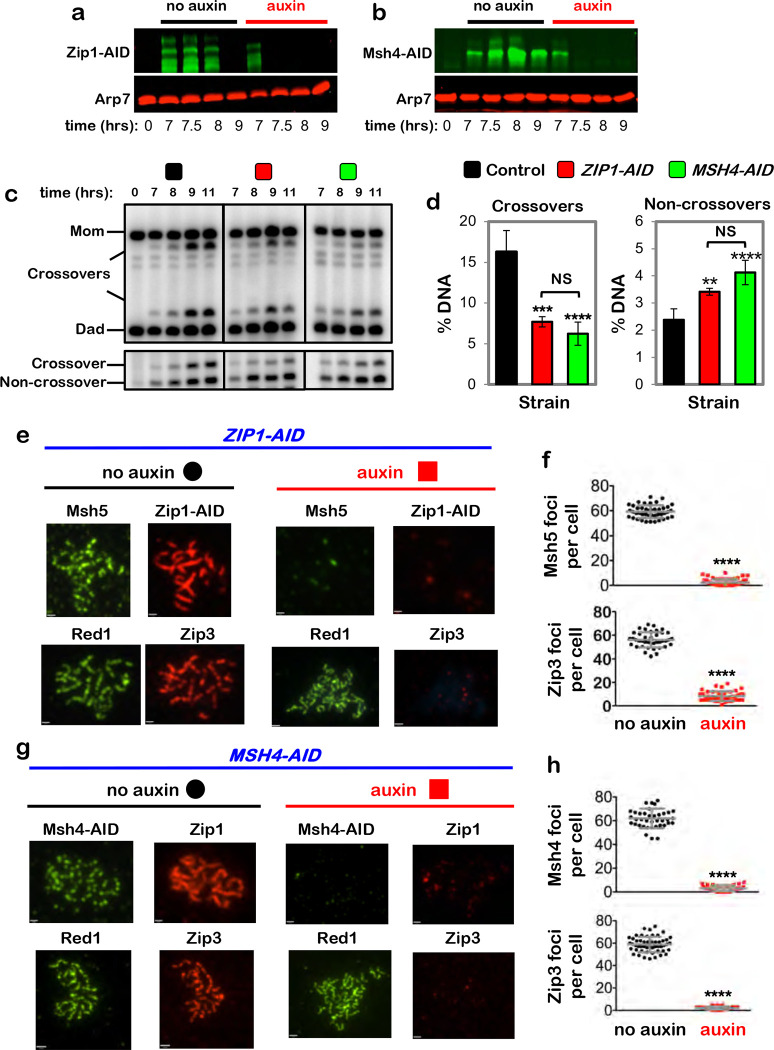
Synaptonemal complex and MutSγ are required for crossover-specific dHJ resolution **a,** Western blot analysis of Zip1-AID from subcultures with or without the addition of auxin at 7 hours. Arp7 is a loading control. **b,** Western blot analysis of Msh4-AID from subcultures with or without the addition of auxin at 7 hours. Arp7 is used as a loading control. **c,** Representative 1D-gel Southern analysis of crossover (upper panel) and non-crossover (lower panel) formation in control, *ZIP1-AID* (with auxin) and *MSH4-AID* (with auxin) strains. **d,** Final levels of crossovers and non-crossovers at 11 hrs from the indicated strains. Error bars represent SD for three independent experiments. Statistical comparisons with control unless indicated. Tukey’s multiple comparisons test, ****P<0.0001, **P=0.0099, NS, not significant, *P*=0.3353 (*ZIP1-AID* vs. *MSH4-AID* for crossover analysis), *P*=0.0763 (*ZIP1-AID* vs. *MSH4-AID* for non-crossover analysis). **e,** Representative images of surface-spread meiotic nuclei from pachytene-arrested *ZIP1-AID* cells with or without the addition of auxin, immunostained for the indicated markers. Scale bars = 1 μm. **f,** Quantification of Msh5 and Zip3 immunostaining foci from the experiments represented in panel **e**. 40–60 nuclei were counted in each case. Error bars represent SD. Unpaired two-tailed *t* test, *P*<0.0001. **g,** Representative images of surface-spread meiotic nuclei from pachytene-arrested *MSH4-AID* cells with or without the addition of auxin, immunostained for the indicated markers. **h,** Quantification of Msh4 and Zip3 immunostaining foci from the experiments represented in panel **g**. 40–60 nuclei were counted in each case. Error bars represent SD. 40–60 nuclei were counting for each set of data. In **f** and **g**, scale bars = 1 μm. Unpaired two-tailed *t* test, ****P<0.0001.

To discern which feature(s) of pachytene chromosomes is important for crossover-specific dHJ resolution, SC component Zip1 and pro-crossover factor MutSγ were inactivated as cells were released from pachytene arrest (Fig. 4a,b), as described in Fig. 1a. Zip1 assembles into the transverse filaments of the SC central region and is essential for synapsis ^25,26^. MutSγ (a complex of Msh4 and Msh5) binds and stabilizes nascent joint molecules to promote homolog synapsis and dHJ formation ^27–29^. Both Zip1-AID and Msh4-AID degradation caused phenotypes similar to those resulting from loss of cohesin, i.e. reduced crossovers and increased noncrossovers (Fig. 4c,d), indicating continued roles for the SC central region and MutSγ after dHJs have formed, to maintain their crossover resolution fate.

**Extended Data Fig. 3.**
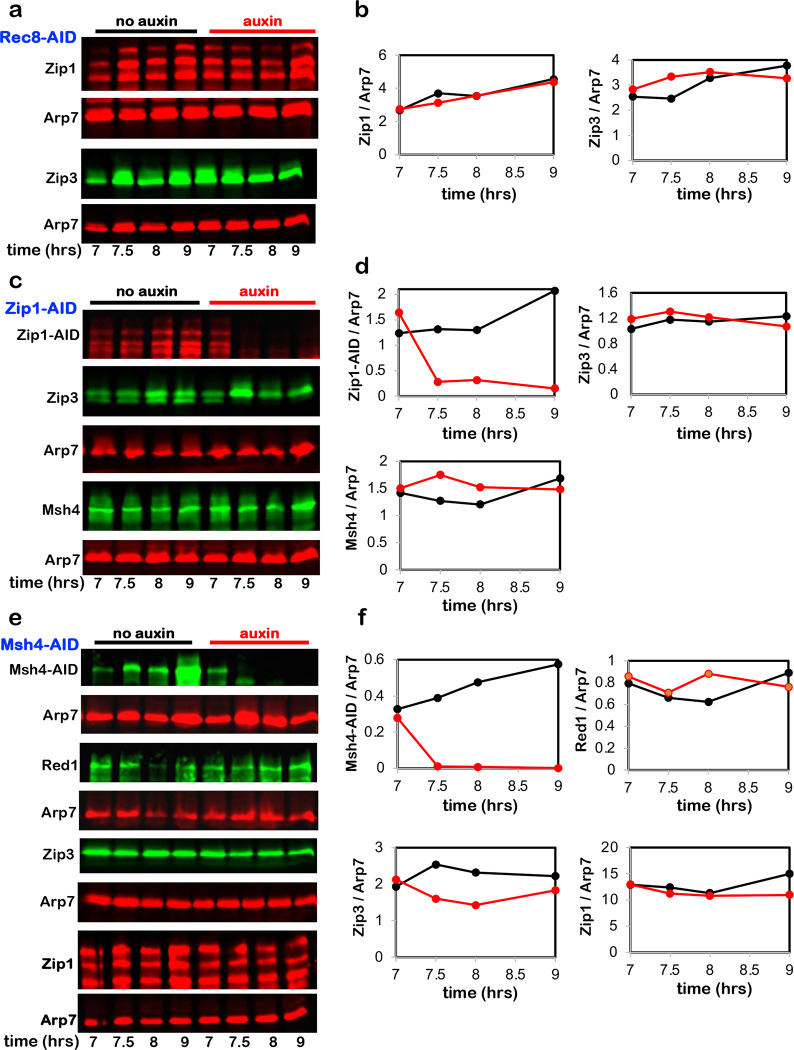
Targeted degradation does not cause off-target effects. **a,** Representative Western blot analysis of Zip1 and Zip3 with or without Rec8-AID degradation following auxin addition at 7 hours. **b,** Quantification of Zip1 and Zip3 level from the experiments shown in **a**. **c,** Representative Western blot analysis of Zip3 and Msh4 with or without Zip1-AID degradation following auxin addition at 7 hours. **d,** Quantification of Zip1-AID, Zip3, and Msh4 levels from the experiments shown in **c**. **e,** Representative Western blot analysis of Red1, Zip3, and Zip1 with or without Msh4-AID degradation following auxin addition at 7 hours. **F,** Quantification of Msh4-AID, Red1, Zip3, and Zip1 level from the experiments shown in **e**. Arp7 is the loading control in all experiments.

This analysis suggests a hierarchical model of pachytene chromosome structure in which cohesin facilitates crossover-specific dHJ resolution by stabilizing the SC, which in turn stabilizes CRCs. This interpretation was tested by immunostaining the chromosomes of pachytene-arrested cells (as described in Fig. 3) following degradation of Zip1-AID (Fig.4e,f) or Msh4-AID (Fig.4g,h), respectively. When Zip1-AID was degraded, homologs desynapsed but axis integrity was maintained as shown by robust Red1 localization (Fig. 4e). As predicted, CRCs marked by Msh5 and Zip3 foci were diminished (Fig. 4e,f). As Zip3 and Msh4 protein levels remained high in these cells (Extended Data Fig. 3), we infer that CRCs are disassembled when Zip1-AID is degraded. Unexpectedly, desynapsis occurred when Msh4-AID was degraded revealing that the maintenance of SCs during pachytene requires the continued presence of CRCs (Fig. 4g). Zip3 foci were also lost when Msh4-AID was degraded highlighting the interdependence between joint-molecule binding (MutSγ) and regulatory (Zip3) components of CRCs (Fig. 4g,h).

## Pachytene chromosome structures protect dHJs from noncrossover resolution by the BLM/STR complex

**Extended Data Fig. 4.**
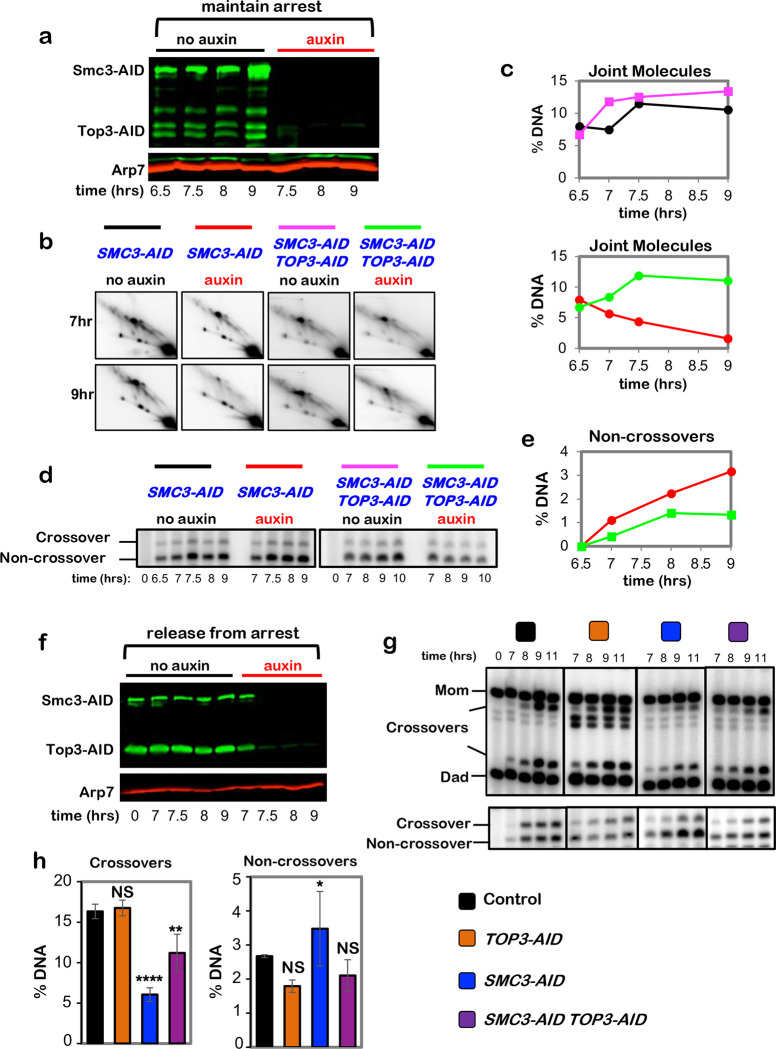
Cohesin protects dHJs from dissolution by the BLM/STR complex. **a,** Western blot analysis of Smc3-AID and Top3-AID co-degradation in pachytene-arrested cells following the addition of auxin at 7 hours. Arp7 is a loading control. **b,** Representative 2D gel Southern analysis of joint molecules from subcultures of pachytene arrested *SMC3-AID* and *SMC3-AID TOP3-AID* strains, with or without the addition of auxin. **c,** Quantification of total joint molecules from experiments shown in **b**. **d,** Representative 1D gel Southern analysis of non-crossover formation from subcultures of pachytene arrested *SMC3-AID* and *SMC3-AID TOP3-AID* strains, with or without the addition of auxin (the same experiments analyzed in panel **b**). **e,** Quantification of non-crossovers from pachytene arrested *SMC3-AID* (red) and *SMC3-AID TOP3-AID* (green) strains following the addition of auxin. **f,** Western blot analysis of Smc3-AID and Top3-AID co-degradation in cells released from pachytene-arrest and addition of auxin at 7 hours. Arp7 is used as loading control. **g,** Representative 1D-gel Southern analysis of crossover (upper panel) and non-crossover (lower panel) formation in control, *TOP3-AID* (with auxin), *SMC3-AID* (with auxin) and *SMC3-AID TOP3-AID* (with auxin) following release from pachytene arrest. **h,** Final levels of crossovers and non-crossovers at 11 hrs from experiments represented in **g** (mean ± SD, 3 (*SMC3-AID* and *TOP3-AID SMC3-AID*) or 4 (*TOP3-AID* and *SMC3-AID*) independent experiments). Statistical comparisons with control: ****P<0.0001, ** *P*= 0.0038, NS, not significant, *P*=0.9876 (crossovers, *TOP3-AID* vs. control), *P=0.0423. NS, not significant, *P*=0.8549 (non-crossovers, *TOP3-AID* vs. control), NS, not significant, *P* >0.9999 (non-crossovers, *TOP3-AID SMC3-AID* vs. control), Dunnett’s multiple comparisons test.

dHJs normally remain stable in pachytene-arrested *NDT80-IN* cells because polo-like kinase Plk1^Cdc5^, which activates their resolution, is not expressed ^12,30^. However, dHJ levels decreased ~3-fold following degradation of Rec8-AID (Fig. 5a,b) implying that pachytene chromosome structures protect dHJs from aberrant resolution by a Plk1^Cdc5^-independent resolution activity. A good candidate for this activity is Sgs1-Top3-Rmi1 (STR), the budding yeast ortholog of human BLM complex BLM-TOPIIIα-RMI1/2, and a robust decatenase enzyme that “dissolves” dHJs specifically into noncrossover products ^7,31–34^. Confirming this prediction, dHJs were stabilized when Rec8-AID and Top3-AID were simultaneously degraded (Fig. 5a–c; co-degrading Smc3-AID and Top3-AID gave very similar results, Extended Data Fig. 4). Moreover, dHJ resolution following Rec8-AID degradation alone produced noncrossover products that did not form when Top3-AID was also degraded (Fig. 5b).

**Extended Data Fig. 5.**
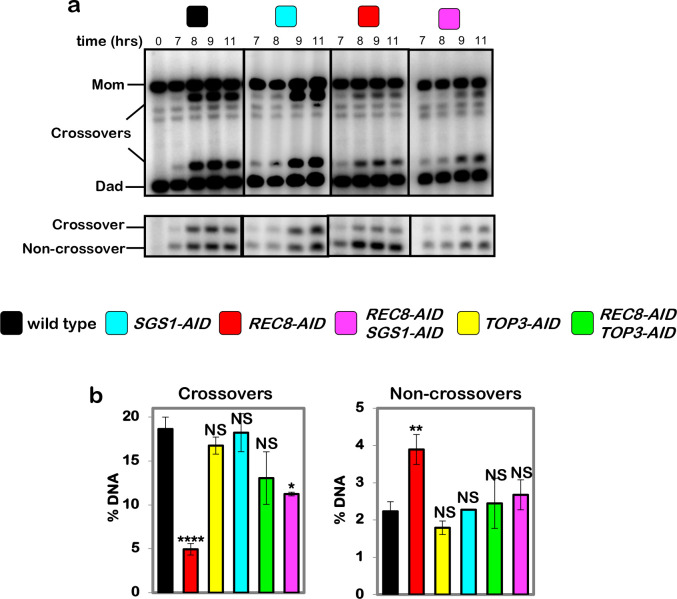
Sgs1-AID degradation partially suppresses the crossover defect resulting from degradation of Rec8-AID. **a,** Representative 1D-gel Southern analysis of crossover (upper panel) and non-crossover (lower panel) formation following release from pachytene arrest in control, *SGS1-AID* (with auxin), *REC8-AID* (with auxin), *REC8-AID SGS1-AID* (with auxin). **b,** Final levels of crossovers and non-crossovers at 11 hrs from the indicated strains (mean ± SD). Data for *TOP3-AID* and *REC8-AID TOP3-AID* from Fig. 5e are shown for comparison. ****P<0.0001, *P=0.0162, **P=0.0012; NS, not significant. Crossovers: *P*=0.9378 (*TOP3-AID* vs. control), *P*=0.3345 (*SGS1-AID* vs. control), *P*=0.1490 (*REC8-AID TOP3-AID* vs. control); Non-crossovers: *P*=0.4759 (*TOP3-AID* vs. control), *P*>0.9999 (*SGS1-AID* vs. control), *P*= 0.9465 (*REC8-AID TOP3-AID* vs. control), *P*=0.5646 (*REC8-AID SGS1-AID* vs. control), Dunnett’s multiple comparisons tests. Number of experiments: control n=3, *TOP3-AID* n=3, *SGS1-AID* n=2, *REC8-AID* n=3, REC8-AID SGS1-AID n=2, *TOP3-AID REC8-AID* n=2.

The stability of dHJs in pachytene-arrested cells following co-degradation of Rec8-AID and Top3-AID suggests that resolution is once again dependent on Plk1^Cdc5^ and the crossover defect resulting from Rec8-AID degradation may be rescued. Indeed, expression of *NDT80-IN* while simultaneously degrading Rec8-AID and Top3-AID resulted in a 2.6-fold increase in crossovers relative to degradation of Rec8-AID alone, while noncrossovers decreased 1.6-fold (Fig. 5d–f; co-degradation of Rec8-AID and Sgs1-AID gave similar results, Extended Data Fig. 5).

**Extended Data Fig. 6.**
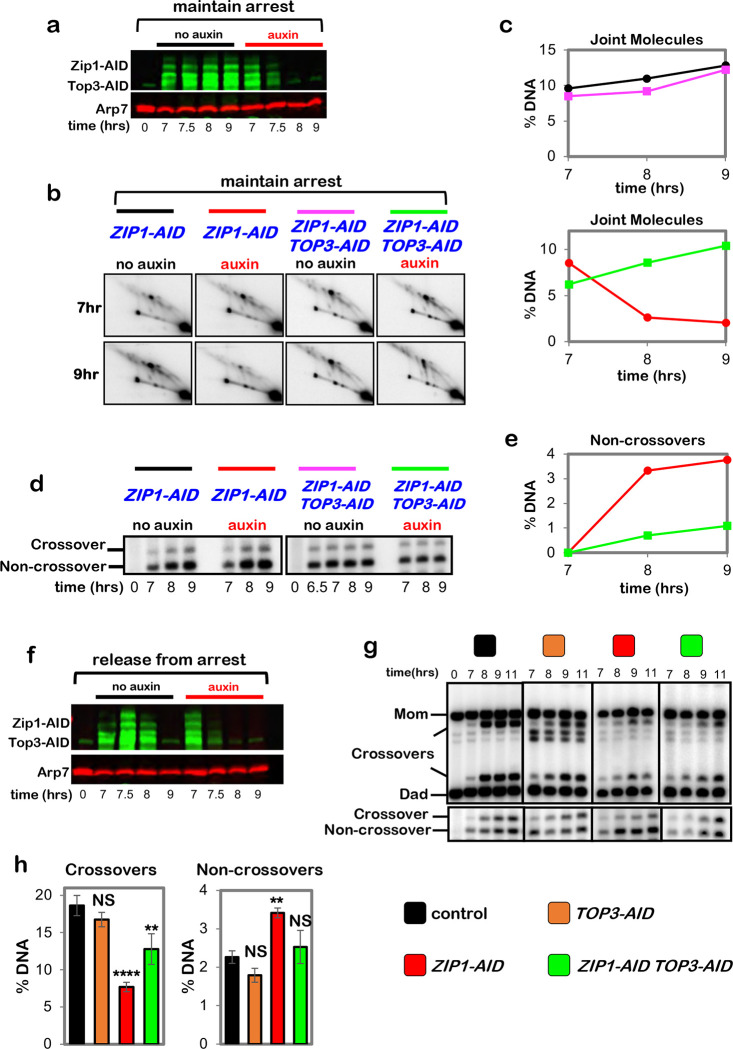
Zip1 protects double-Holliday junctions from aberrant resolution mediated by the BLM/STR complex. **a,** Western blot analysis of Zip1-AID and Top3-AID co-degradation in pachytene-arrested cells following the addition of auxin at 7 hours. Arp7 is a loading control. **b,** Representative 2D gel Southern analysis of joint molecules from subcultures of pachytene arrested *ZIP1-AID* and *ZIP1-AID TOP3-AID* strains, with or without the addition of auxin. **c,** Quantification of total joint molecules from experiments shown in **b**. **d,** Representative 1D gel Southern analysis of non-crossover formation from subcultures of pachytene arrested *ZIP1-AID* and *ZIP1-AID TOP3-AID* strains, with or without the addition of auxin (the same experiments analyzed in panel **b**). **e,** Quantification of non-crossovers from pachytene arrested *ZIP1-AID* (red) and *ZIP1-AID TOP3-AID* (green) strains following the addition of auxin. **f,** Western blot analysis of Zip1-AID and Top3-AID co-degradation in cells released from pachytene-arrest and addition of auxin at 7 hours. **g,** Representative 1D-gel Southern analysis of crossover (upper panel) and non-crossover (lower panel) formation in control, *TOP3-AID* (with auxin), *ZIP1-AID* (with auxin) and *ZIP1-AID TOP3-AID* (with auxin) following release from pachytene arrest. **h,** Final levels of crossovers and non-crossovers at 11 hrs from experiments represented in **g** (mean ± SD). Statistical comparisons with control: ****P<0.0001, **P=0.0022, NS, not significant. *P*=0.1011 (crossovers *TOP3-AID* vs. control), *P*=0.1104 (non-crossovers *TOP3-AID* vs. control), *P*=0.5171 (non-crossovers *ZIP1-AID TOP3-AID* vs. control), Dunnett’s multiple comparisons tests. Number of experiments: control: n=3, *TOP3-AID*: n=3, *ZIP1-AID*: n=2, *ZIP1-AID TOP3-AID*: n=2.

**Extended Data Fig. 7.**
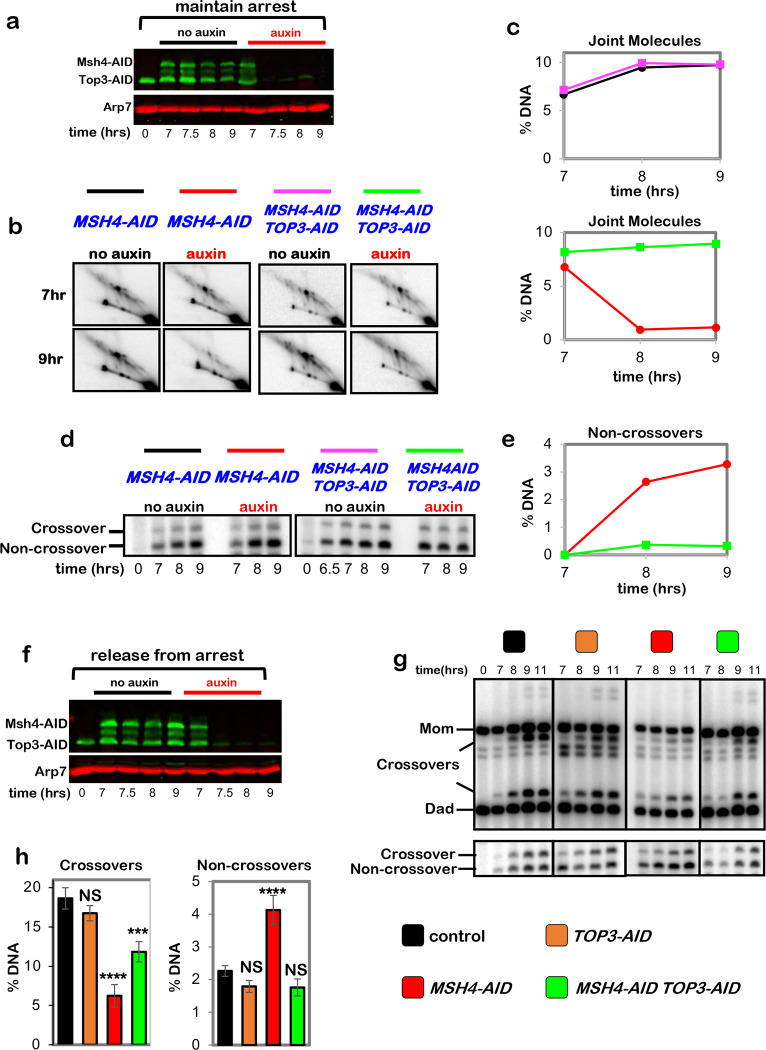
MutSγ protects double-Holliday junctions from aberrant resolution mediated by the BLM/STR complex. **a,** Western blot analysis of Msh4-AID and Top3-AID co-degradation in pachytene-arrested cells following the addition of auxin at 7 hours. Arp7 is a loading control. **b,** Representative 2D gel Southern analysis of joint molecules from subcultures of pachytene arrested *MSH4-AID* and *MSH4-AID TOP3-AID* strains, with or without the addition of auxin. **c,** Quantification of total joint molecules from experiments shown in **b**. **d,** Representative 1D gel Southern analysis of non-crossover formation from subcultures of pachytene arrested *MSH4-AID* and *MSH4-AID TOP3-AID* strains, with or without the addition of auxin (the same experiments analyzed in panel **b**). **e,** Quantification of non-crossovers from pachytene arrested *MSH4-AID* (red) and *MSH4-AID TOP3-AID* (green) strains following the addition of auxin. **f,** Western blot analysis of Msh4-AID and Top3-AID co-degradation in cells released from pachytene-arrest and addition of auxin at 7 hours. **g,** Representative 1D-gel Southern analysis of crossover (upper panel) and non-crossover (lower panel) formation in control, *TOP3-AID* (with auxin), *MSH4-AID* (with auxin) and *MSH4-AID TOP3-AID* (with auxin) following release from pachytene arrest. **h,** Final levels of crossovers and non-crossovers at 11 hrs from experiments represented in **g** (mean ± SD). Statistical comparisons with control: ****P<0.0001, ***P=0.0003, NS, not significant, *P*>0.9999 (crossovers, *TOP3-AID* vs. control), *P*=0.0821 (non-crossovers *TOP3-AID* vs. control), *P*=0.0638 (non-crossovers *TOP3-AID MSH4-AID* vs. control), Dunnett’s multiple comparisons test. Number of experiments: control: control: n=6, *TOP3-AID*: n=3, *MSH4-AID*: n=6, *TOP3-AID MSH4-AID*: n=3.

Moreover, stabilization of dHJs and partial rescue of the crossover defects resulting from Zip1-AID or Msh4-AID degradation were also seen when Top3-AID was simultaneously degraded (Extended Data Figs. 6 and 7).

**Extended Data Fig. 8.**
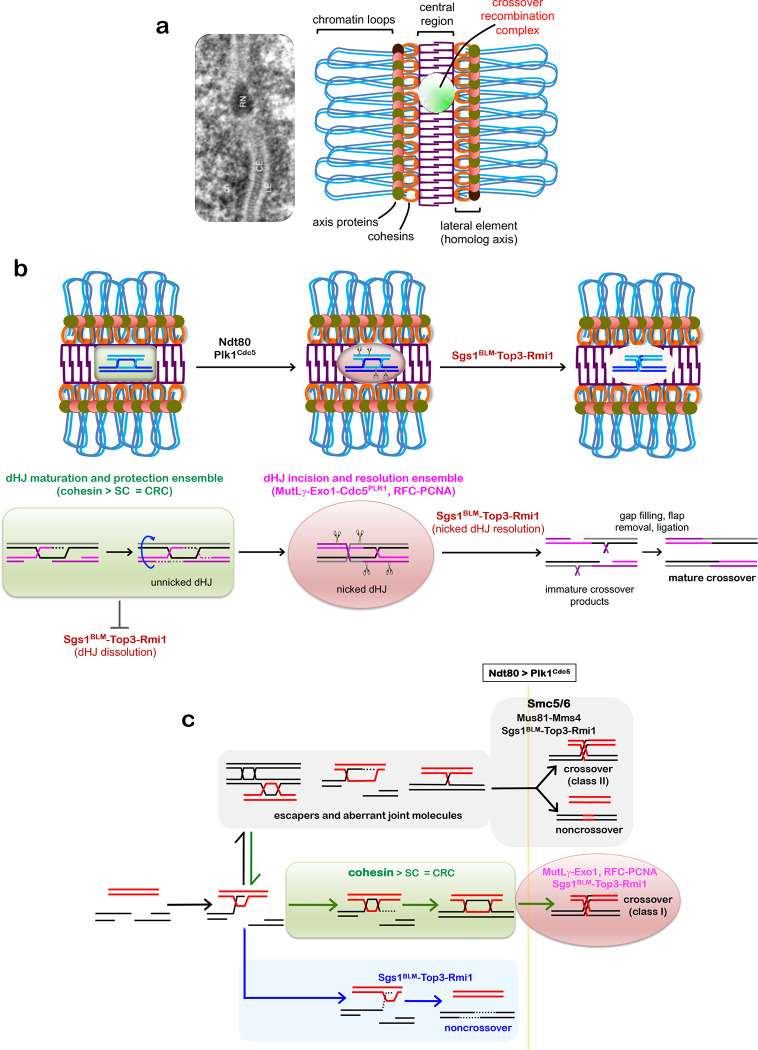
Summary and Model. **a,** Electron micrograph of SC from *Blaps cribrosa*^56^ and schematic highlighting the key features of pachytene-stage chromosomes. Ch, chromatin; LE, lateral element; CE, central element, RN, recombination nodule (the site of a crossover recombination complex, CRC). **b,** Cohesin, SC and CRCs constitute a dHJ maturation and protection ensemble that facilitates dHJ formation and protects unnicked dHJs from being dissolved into non-crossovers by the BLM/STR complex. dHJ protection must be maintained until Ndt80-dependent expression of polo-like kinase Plk1^Cdc5^ triggers crossover-specific resolution via a two-step mechanism: (i) strand-specific nicking of dHJs by the MutLγ endonuclease and associated factors^4^; (ii) resolution by the BLM/STR complex. Placement of the nicks, on both sides of the two Holliday junctions but distal to the exchange points, specifies a crossover outcome. **c,** Pathways of joint-molecule resolution during meiotic recombination. Resected DSB ends undergo homology search and DNA strand exchange to form nascent displacement loops (D-loops) as common precursors to all pathways. Non-crossover fated D-loops are unwound by BLM/STR to effect synthesis-dependent strand annealing, independently of Plk1^Cdc5^. D-loops that are designated a crossover fate mature into dHJs, facilitated and protected by cohesion-dependent chromosome structures, i.e. SC and CRCs. In this class-I pathway, Plk1^Cdc5^-depenent two-step resolution via MutLγ nicking and BLM/STR disassembly specifies a crossover outcome (as described in **b**). All other joint-molecule structures are resolved via a Smc5/6-dependent pathway that is also dependent on Plk1^Cdc5^ and yields class-II crossovers and non-crossovers.

We conclude that the key facets of pachytene chromosomes – cohesin-based homolog axes, SCs, and CRCs – protect crossover-designated dHJs from being aberrantly dissolved into noncrossover products by the STR complex (see Extended Data Fig. 8b).

## Interdependencies between dHJs, cohesin, synaptonemal complex, and crossover recombination complexes

**Fig. 6.**
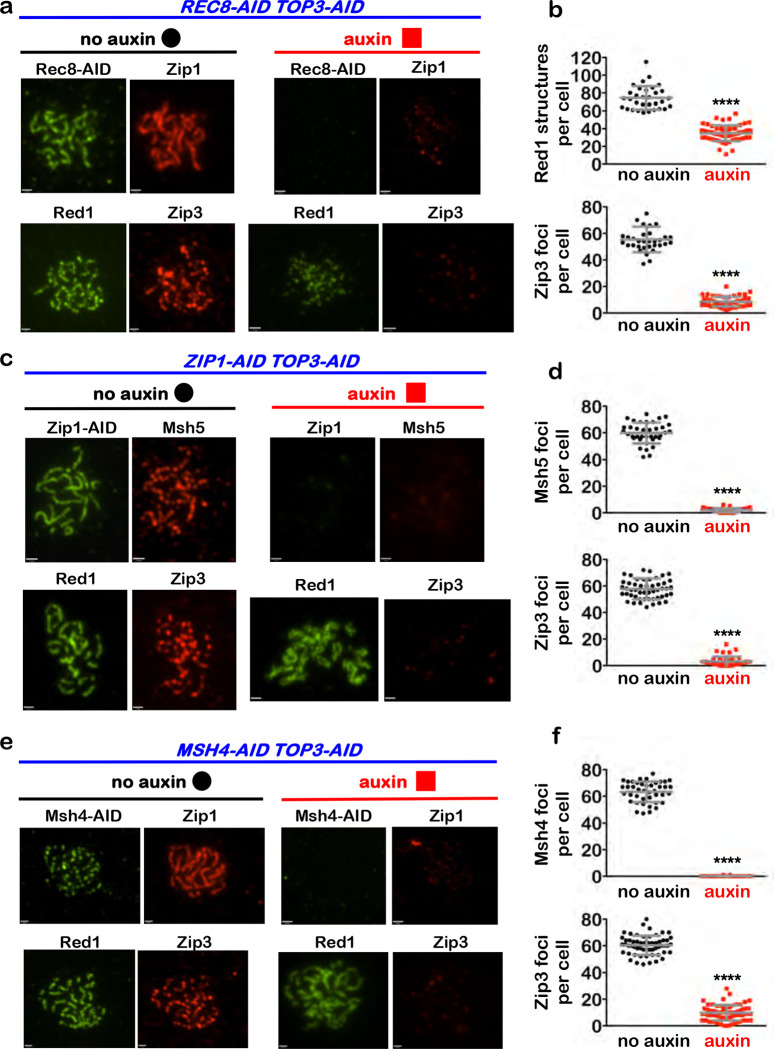
Stabilizing dHJs in pachytene does not bypass interdependence between cohesin, synapsis, and crossover recombination complexes **a,** Representative images of surface-spread meiotic nuclei from pachytene-arrested *REC8-AID TOP3-AID* cells with or without the addition of auxin, immunostained for the indicated markers. **b,** Quantification of Red1 and Zip3 immunostaining structures from the experiments represented in panel **a**. **c,** Representative images of surface-spread meiotic nuclei from pachytene-arrested *ZIP1-AID TOP3-AID* cells with or without the addition of auxin, immunostained for the indicated markers. **d,** Quantification of Msh5 and Zip3 immunostaining foci from the experiments represented in panel **c**. **e,** Representative images of surface-spread meiotic nuclei from pachytene-arrested *MSH4-AID TOP3-AID* cells with or without the addition of auxin, immunostained for the indicated markers. **f,** Quantification of Msh4 and Zip3 immunostaining foci from the experiments represented in panel **e**. Scale bars = 1 μm. Error bars represent SD. 40–60 nuclei were counted in each case. Unpaired two-tailed *t* test, ****P<0.0001.

We wondered whether stabilizing dHJs also rescues the cytological defects resulting from degradation of Rec8-AID, Zip1-AID, or Msh4-AID. To this end, Top3-AID was co-degraded together with Rec8-AID (Fig. 6a,b), Zip1-AID (Fig. 6c,d) or Msh4-AID (Fig. 6e,f) in pachytene-arrested cells, and chromosomes were immunostained for markers of synapsis (Zip1), and CRCs (Msh4 and Zip3). In each case, cytological phenotypes were largely indistinguishable from those observed when Rec8-AID, Zip1-AID, or Msh4-AID were degraded alone. SCs and CRCs disassembled following co-degradation of Rec8-AID and Top3-AID (Fig. 6a,b); CRCs dissociated when Zip1-AID and Top3-AID were co-degraded (Fig. 6c,d); and SCs disassembled following co-degradation of Msh4-AID and Top3-AID (Fig. 6e,f). Thus, maintaining inter-homolog connections by stabilizing dHJs does not bypass the interdependencies between cohesin, SC, and crossover recombination complexes.

## Discussion

The SC is an ancient structure that evolved with the emergence of sexual reproduction. Several functions are attributed to the SC: through pairwise synapsis of homologs, it generates topological order in the nucleus; synapsis also attenuates formation of DSBs, facilitates their repair, and dampens associated checkpoint signaling; and at designated crossover sites, SC facilitates dHJ formation^5^. This study reveals that SC also enables crossover-specific resolution by protecting dHJs from unscheduled resolution into noncrossovers by the conserved BLM/STR complex (Extended Data Fig. 8). Failure of this critical final step of meiotic recombination will result in unlinked univalent chromosomes that are prone to missegregation resulting in aneuploid gametes that are associated with infertility, miscarriage, and congenital disease in humans.

Our data indicate that dHJs formed at crossover-designated rombination sites do not possess an intrinsic structure or topology that constrains their resolution fate to ensure crossing over. We previously proposed a model of crossover-specific resolution in which asymmetric loading of PCNA during the DNA synthesis associated with dHJ formation directs strand-specific nicking by the MutLγ endonuclease on both sides of the two Holliday junctions, but distal to the exchange points^4^. This pattern of incisions always specifies a crossover outcome when the nicked dHJs are disassembled by the BLM/STR complex. This two-step resolution model reconciles the cryptic pro-crossover role of BLM/STR during meiosis, which sharply contrasts its well-characterized anti-crossover role when dissolving unnicked dHJs into noncrossver products (Extended Data Fig. 8b).

Within this framework, we propose that mature dHJs are unnicked and therefore vulnerable to dissolution until MutLγ is activated via Ndt80-dependent expression of Plk1^Cdc5^. By preventing BLM/STR from resolving dHJs until they have been nicked by MutLγ, the SC imposes the sequential steps required for crossover-specific resolution (Extended Data Fig. 8b).

We suggest that the SC protects dHJs by stabilizing CRCs, components of which can directly bind HJs (including MutSγ, MutLγ, Zip2^SHOC1^-Spo16, Mer3^HFM1^)^23,35^ and may directly compete with BLM/STR for binding dHJs and/or constrain its activity. Indeed, components of the SC-central region help recruits and stabilize CRCs ^28,36–39^. Moreover, *C. elegans* SC-central region proteins assemble transient crossover-specific compartments or “bubbles” that may protect CRCs until crossover-specific resolution is executed^37^.

Conversely, we found that CRCs are required to maintain synapsis during pachytene. This observation is consistent with studies indicating that the SC central region is initially dynamic and labile but becomes more stable, contingent on the development of CRCs^40–43^. Intriguingly, new subunits are continually incorporated into the SC central region after synapsis^40,43^; and in budding yeast, incorporation of new Zip1 molecules occurs predominantly at CRC sites^40^. Given that Zip1 appears to recruit CRC components directly^38,44^, this suggests a possible mechanism for the mutual stabilization of SCs and CRCs. This relationship may result in SCs with non-uniform structure and stability^40^, which could influence resolution fates and explain why sites of crossing over are the last to desynpase during diplotene^45^.

The interdependence and dynamicity of SCs and CRCs will render chromosomal interactions readily reversible until dHJs are resolved into crossovers. These attributes could help adjust and proof-read homologous synapsis, minimize and resolve synaptic interlocks^46^, and preserve genome stability by destabilizing interactions between non-allelic and diverged sequences.

Our analysis also reveals that two SMC complexes mediate essentially all Plk1^Cdc5^-dependent joint-molecule resolution during meiosis (Extended Data Fig. 8c). Rec8-cohesin is required to maintain SCs and CRCs, thereby protects dHJs to facilitates crossover-specific dHJ resolution via MutLγ and BLM/STR. Whether local or global functions of Rec8-cohesin are important for crossover-specific dHJ resolution, and the roles of cohesive versus chromatin-loop organizing populations of Rec8-cohesin remain unclear. An independent Smc5/6 pathway is essential for resolution by Mus81-Mms4^EME1^ and BLM/STR, producing a mixture of crossovers and noncrossovers. Despite these distinctions, Rec8-cohesin and Smc5/6 could have common functions to constrain favorable joint-molecule topology^47^ and control access by resolving enzymes.

## Methods

### Data reporting

No statistical methods were used to predetermine sample size. The experiments were not randomized, and the investigators were not blinded to allocation during experiments. Blinding was employed during outcome assessment for cytology experiments.

### Yeast Strains

For full genotypes see Supplementary Information Table 1. The AID system ^11^ was optimized for meiosis by replacing the promoter of the *P*_*ADH1*_*-OsTIR1* cassette with the *CUP1* promoter^32^. C-terminal fusion of a minimal AID degron to targeted proteins was constructed using plasmid pHyg-AID*−9Myc as template for PCR-mediated allele replacement ^48^. To construct an internal degron allele of *ZIP1,* AID degron sequences were inserted into plasmid pMPY-3xHA and integrated after codon 700 via PCR epitope tagging ^49^. Primers used to construct AID degron alleles are listed in Supplementary Information Table 2. The estrogen-inducible *IN-NDT80 GAL4-ER* system has been described ^50–52^.

### Meiotic Time Courses and DNA Physical Assays

Detailed protocols for meiotic time courses and DNA physical assays at the *HIS4::LEU2* locus have been described ^53^. At 6.5 h after induction of meiosis, CuSO4 (100 mM stock in dH2O) was added for a final concentration of 50 μM to induce expression of *P*_*CUP1*_*-OsTIR1* (encoding the Tir1 E3 ligase) and cell cultures were split. At 7 h, estradiol (5 mM stock, Sigma E2758 in ethanol) was added for a final concentration of 1 μM to both subcultures to induce *NDT80-IN*. Simultaneously, auxin (3-indoleacetic acid, Sigma 13750, 2 M stock in DMSO) was added to one subculture for a final concentration of 2 mM; an equivalent volume of DMSO was added to the no-auxin control subculture. At 7.5 h, auxin was added again at 1 mM. To analyze the timing and efficiency of meiotic divisions and sporulation, cells were fixed in 40% ethanol, 0.1 M sorbitol, stained with DAPI, and ~200 cells were categorized for each time point. For imaging, DAPI-stained cells were mounted in antifade (Vectashield, Vector Laboratories, Inc.) and digital images captured using a Zeiss AxioPlan II microscope, Hamamatsu ORCA-ER CCD camera and Volocity software.

### Chromosome spreading and immunofluorescence microscopy

Cell samples were collected and processed for chromosome spreading and immunostaining essentially as described ^54^. Primary antibodies kindly provided by Akira Shinohara were chicken anti-Red1 (1:500 dilution), rabbit anti-Msh5 (1:750), and rabbit anti-Zip3 (1:500); anti-Zip1 (1:400) was a gift from Scott Keeney; anti-myc (1:1000, Roche 11667149001) was used to detect AID-9myc fused proteins. All primary antibodies were incubated overnight at room temperature in 100 μl TBS/BSA buffer (10 mM Tris pH 7.5, 150 mM NaCl, 1% BSA). Secondary antibodies anti-rabbit 568 (A11036 Molecular Probes, 1:1,000), anti-mouse 488 (A11029 Molecular Probes, 1:1,000), anti-rabbit 647 (A21245 Invitrogen), and anti-guinea pig 555 (A21435 Life Technologies) were incubated for 1 h at 37 °C. Coverslips were mounted with Prolong Gold antifade reagent (Invitrogen, P36930). Digital images were captured using a Zeiss Axioplan II microscope, Hamamatsu ORCA-ER CCD camera and analyzed using Volocity software. Scatterplots were generated using the GraphPad program in Prism.

### Western Blot Analysis

Whole cell extracts were prepared using a TCA extraction method, essentially as described ^55^. Samples were analyzed by standard SDS-PAGE and Western blotting using the following primary antibodies: anti-c-Myc (1:1000, Roche 11667149001), anti-HA (1:1000, Sigma11583816001), Arp7 (1:10,000, Santa Cruz SC-8960), anti-Msh4 (1:500), and anti-Msh5 (1:500, Msh4 and Msh5 antibodies were a gift from Dr. Akira Shinohara). Secondary antibodies (1:5000) were IRDye^®^ 800CW Donkey anti-Mouse IgG (LI-COR 925–32212), IRDye^®^ 680LT Donkey anti-Goat IgG (LI-COR 925–68024), IRDye^®^ 680LT Donkey anti-Rabbit IgG (LI-COR 925–68023) and IRDye^®^ 800CW Donkey anti-Rabbit IgG (LI-COR 925–32213). Western blots were imaged on an Odyssey Infrared Imager (LI-COR) and quantification of protein bands was performed using Image Studio Lite Ver 4.0 software.

### Statistical analysis and Reproducibility

Statistical analyses were performed using Prism (GraphPad software Inc.). For bar graphs and scatter plots comparing two samples (aggregated from three or more replicate experiments), unpaired *t*-tests were performed. For multiple comparisons, one-way ANOVA was performed (Tukey or Dunnett tests, depending on the specific comparisons being made). For scatter plots and bar graphs, error bars show the mean value with standard deviation. Western blots are representative of at least three repeats (Figs. 1b, 4a 4b, Fig. 5c and 5d; and Extended Data Figs. 1a, 2a, 3a, 3c, 3e, 4a, 4g, 6a, 6f, 7a, 7g). Representative immunofluorescence images were chosen from over 60 samples analyzed in each of at least three biological replicates (Figs. 1e, h, 2a, 3c, 2e, 2g, 3a, 4e 4g, 5a, 5e, 6a, 6c and 6e).

## Extended Data

## Supplementary Material

Supplement 1

## Figures and Tables

**Fig, 5.**
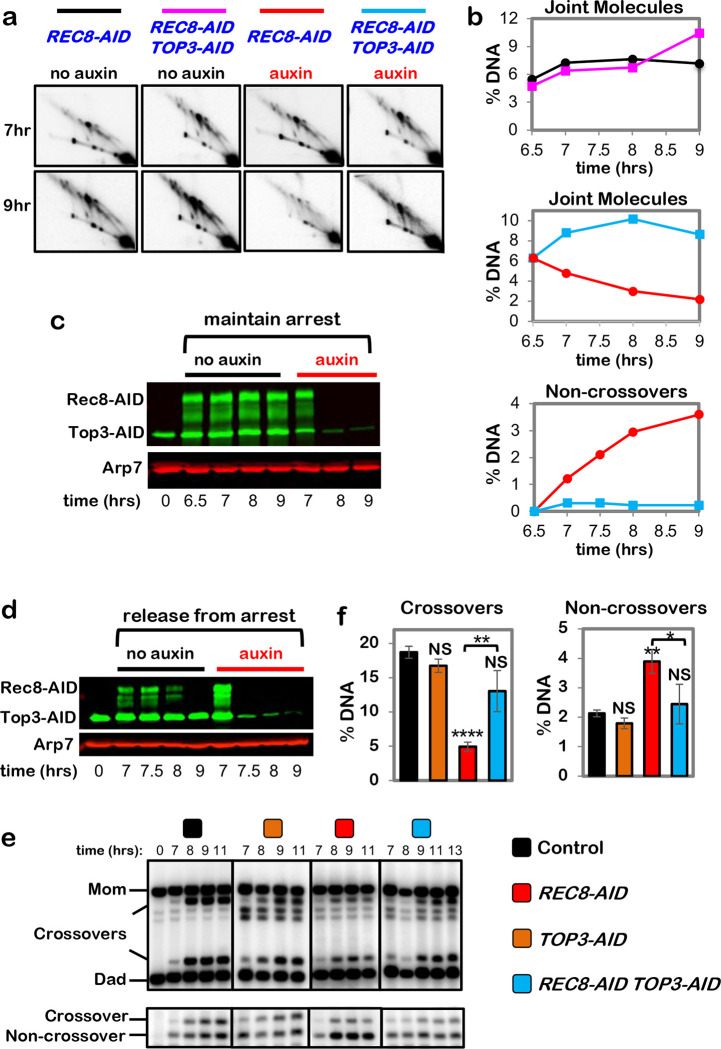
Rec8-cohesin protects double-Holliday junctions from aberrant resolution mediated by the BLM/STR complex. **a,** Representative 2D gel Southern analysis of joint molecules from control, *REC8-AID* (with auxin), *REC8-AID TOP3-AID* (with auxin), and *REC8-AID NSE4-AID* (with auxin) strains. **b,** Quantification of total joint molecule levels from the indicated strains. **c,** Western blot analysis of Top3-AID and Rec8-AID from subcultures with or without the addition of auxin at 7 hours while maintaining pachytene arrest. Arp7 is a loading control. The degradation-resistant fraction of Top3-AID is mitochondrial. **d,** Western blot analysis of Top3-AID and Rec8-AID from subcultures with or without the addition of auxin at 7 hours with release from pachytene arrest. **e,** Representative 1D-gel Southern analysis of crossover (upper panel) and non-crossover (lower panel) formation in control, *TOP3-AID* (with auxin), *REC8-AID* (with auxin) and *REC8-AID TOP3-AID* (with auxin) strains. **f,** Final levels of crossovers and non-crossovers at 11 hrs from experiments represented in panel **e**. Error bars represent SD for three independent experiments. Statistical comparisons with control unless indicated. Tukey’s multiple comparisons test, ****P<0.0001, **P=0.0011 (*REC8-AID* vs. *REC8-AID TOP3-AID* for crossover comparison), NS, not significant, *P*=0.8881 (control vs. *TOP3-AID* for crossover comparison), NS, not significant, *P*=0.0.1516 (control vs. *REC8-AID TOP3-AID* for crossover comparison), **P=0.0041 (control vs. REC8-AID for non-crossover comparison), *P=0.0156, NS, not significant, *P*=0.5131 (control vs. *TOP3-AID* for non-crossover comparison), *P*= 0.9200 (control vs. *REC8-AID TOP3-AID* for non-crossover comparison).

## Data Availability

Relevant data generated or analyzed during this study are included in this Article and its Supplementary Information files. Biological materials are available from the corresponding author.
